# Gypenosides Synergistically Enhances the Anti-Tumor Effect of 5-Fluorouracil on Colorectal Cancer In Vitro and In Vivo: A Role for Oxidative Stress-Mediated DNA Damage and p53 Activation

**DOI:** 10.1371/journal.pone.0137888

**Published:** 2015-09-14

**Authors:** Lulu Kong, Xiaobing Wang, Kun Zhang, Wenjuan Yuan, Qiwen Yang, Jianping Fan, Pan Wang, Quanhong Liu

**Affiliations:** Co-Innovation Center for Qinba Regions’ Sustainable Development, College of Life Sciences, Shaanxi Normal University, Shaanxi, China; Peking University Health Science Center, CHINA

## Abstract

**Objective:**

5-Fluorouracil (5-Fu) has been widely used as a first-line drug for colorectal cancer (CRC) treatment but limited by drug resistance and severe toxicity. The chemo-sensitizers that augment its efficiency and overcome its limitation are urgently needed. Gypenosides (Gyp), the main components from *Gynostemma pentaphyllum* (Thunb.) Makino, has shown potential anti-tumor property with little side-effect. Here, we carefully explored the chemo-sensitization of Gyp to potentiate the anti-tumor effect of 5-Fu *in vitro* and *in vivo*.

**Methodology / Principal Findings:**

3-(4, 5-dimethylthiazol-2-yl)-2, 5-diphenyltertrazolium bromide tetrazolium assay and colony formation test reveal that Gyp could significantly enhance the 5-Fu-caused SW-480,SW-620 and Caco2 cells viability loss. Calcusyn analysis shows that Gyp acts synergistically with 5-Fu. Annexin V-PE/7-AAD staining indicates 5-Fu + Gyp could induce SW-480 cell apoptosis. The activations of caspase 3, caspase 9 and poly (ADP-ribose) polymerase (PARP) were involved in the process. Gyp was also found to up-regulate 5-Fu-caused phospho-p53 expression and thus augment 5-Fu-induced G0/G1 phase arrest. Gyp elevated intracellular ROS level, significantly enhanced 5-Fu-triggered DNA damage response as evidenced by flow cytometry, comet assay and the expression of Ser139-Histone H2A.X. Inhibition of ROS and p53 respectively reversed the cell death induced by 5-Fu + Gyp, suggesting the key roles of ROS and p53 in the process. Moreover, 5-Fu and Gyp in combination exhibits much superior tumor volume and weight inhibition on CT-26 xenograft mouse model in comparison to 5-Fu or Gyp alone. Immunohistochemistry analysis suggests the combinations greatly suppressed tumor proliferation. Preliminary toxicological results show that 5-Fu + Gyp treatment is relatively safe.

**Conclusions:**

As a potential chemo-sensitizer, Gyp displays a splendid synergistic effect with 5-Fu to inhibit cancer cell proliferation and tumor growth. By using 5-Fu and Gyp in combination would be a promising therapeutic strategy for CRC treatment.

## Introduction

Colorectal cancer (CRC) is the third most common malignant cancer and the fourth leading cause of cancer-related deaths worldwide, that accounting for roughly 1.2 million new cases and 600 000 deaths per year [1]. Surgical resection, followed by chemo- or radiotherapy, remains the only established curative treatment for CRC, but limited by drug resistance and severe toxicity [2].

5-Fluorouracil (5-Fu), an analog of uracil, is the most widely used chemotherapeutic drug for treatment solid tumors, especially for colorectal cancer [3]. In tumor cells, 5-Fu is converted to fluorodeoxyuridine monophosphate (FdUMP) to exerting its cytotoxic effect. FdUMP can mis-incorporate into DNA or RNA, inhibit activities of thymidylate synthetase (TS), ultimately cause cell apoptosis [4]. Nonetheless, the overall response rate with 5-Fu monotherapy as first-line treatment for CRC is quite limited (approximately10–20%) [5]. Further increased dosage would produce drug resistance and unavoidable side effects thus limits its therapeutic effect. Recently, combination or multi-component (rather than single-agent) therapy, in which two or more kinds of drugs are used simultaneously, is a proved treatment for cancer [6,7]. 5-Fu combined with new cytotoxic drugs such as oxaliplatin, resveratrol and irinotecan have not only improved the response rates to 40–50%, but also reduced the undesirable reaction of these drugs [8–10], suggesting that 5-Fu has a favorable safety profile and may be suitable for combination treatment with other drugs. Despite these improvements, new therapeutic strategies and new chemo-sensitizers are urgently needed.

**Fig 1:**
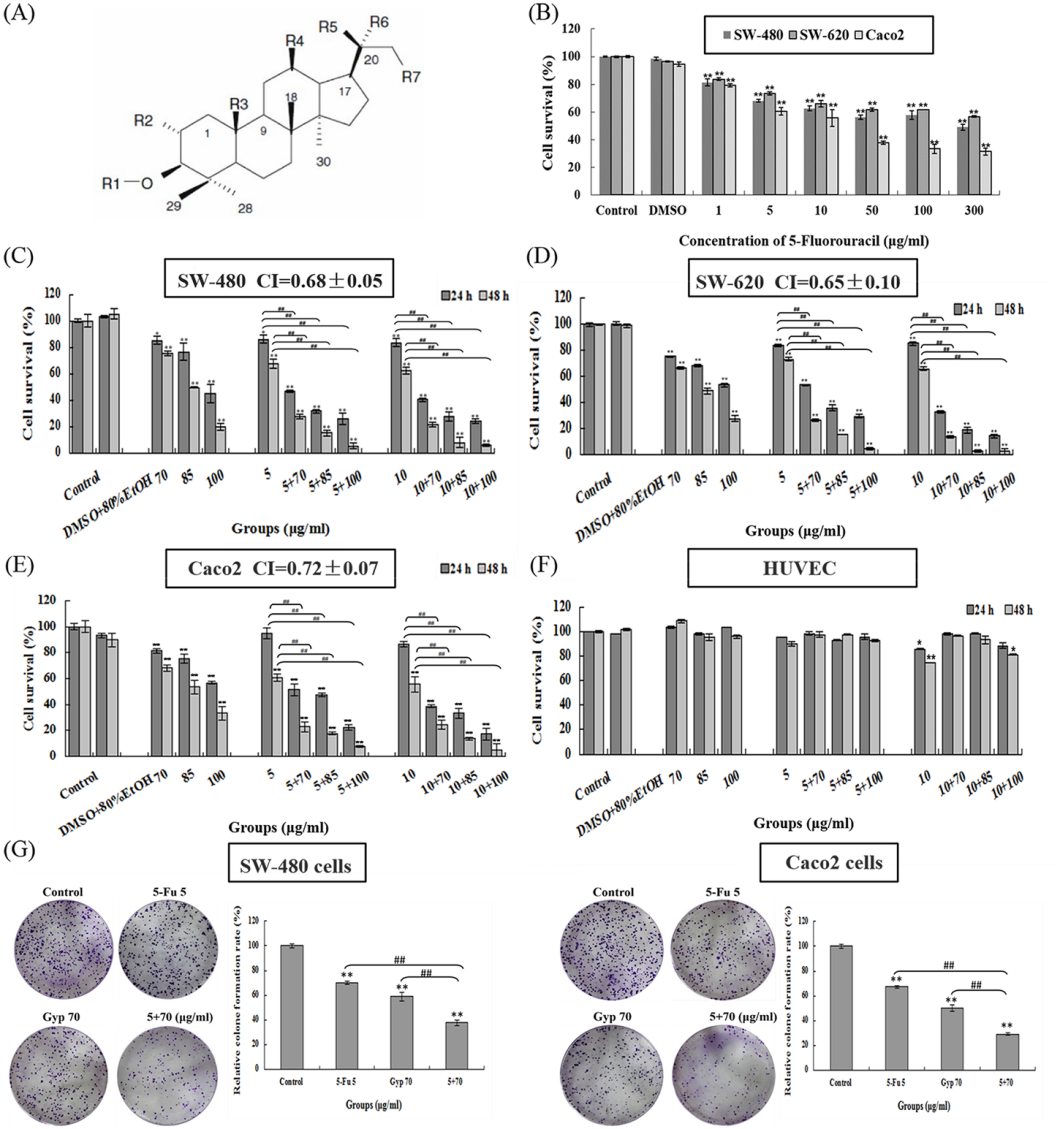
Gyp potentiates 5-Fu-induced cell proliferation inhibition. (A) Chemical dammarane skeleton structure of Gypenosides. (B) Cell viability was measured by MTT assay at 48 h after 5-Fu treatment in SW-480,SW-620 and Caco2 cells. (C-F) Cell viability was measured by MTT assay at 24 and 48 h after 5-Fu and / or Gyp treatment in SW-480,SW-620 and Caco2 cells. Combination index (CI) value was analyzed using CalcuSyn software. CI < 1 indicates a synergistic effect. (G) Colony formation of SW-480 and Caco2 cells after 5-Fu and / or Gyp treatment. All data are expressed as means ± SD of triplicates and p* < 0.05, p** < 0.01 versus control; *p* ^*##*^ < 0.01 versus 5-Fu or Gyp alone group.

Gypenosides (Gyp), the major components extracts from *Gynostemma pentaphyllum* (Thunb.) Makino, exists mainly as dammarane-type triterpene glycosides (Fig 1A) and has been used as a traditional popular folk medicine in Asia for centuries to treat cancer [11–13], hyperlipoproteinemia [14], hepatitis [15], and cardiovascular disease [16]. Experimental study has reported that Gyp could trigger apoptosis in human colorectal cancer 205 cells through mitochondria-dependent pathway and activation of caspase-3 [17]. Our previous investigations also suggest that Gyp inhibited human colorectal cancer SW-480 and SW-620 cells proliferation and migration in a dose- and time-dependent manner [18,19]. Despite these potencies, Gyp was relatively less toxic to human normal cells [20], showing potential application in cancer therapy. However, there is no any information about the chemo-sensitization effect of Gyp until now. And whether Gyp can become a good chemo-sensitizer to amplify the effectiveness of chemotherapy in clinic is not clear. In the present study, we utilize the human colorectal cancer SW-480,SW-620,Caco2 cells and CT-26 xenograft mouse model to explore the possible chemo-sensitization effect of Gyp to potentiate the anti-tumor effect of 5-Fu *in vitro* and *in vivo*. To the best of our knowledge, the present study is the first *in vitro* and *in vivo* preclinical research that assesses the chemo-sensitization effect of Gyp and the anti-tumor effect of using 5-Fu and Gyp in combination. These findings may provide a new therapeutic strategy to achieve anti-cancer synergism.

## Materials and Methods

### Chemicals and reagents

Gypenosides (Gyp) was kindly provided by Ankang Pharmaceutical Institute of the Beijing University (Shaanxi, China) and dissolved in 80% ethanol (EtOH) to a final storage concentration of 100 mg/ml. 5-Fluorouracil (5-Fu) was purchased from Sigam-Aldrich (St. Louis, Mo, USA) and dissolved in dimethyl sulfoxide (DMSO) also to a final storage concentration of 100 mg/ml. Gyp and 5-Fu solution were sterilized through 0.22μm filter for use in subsequent experiments and stored in -20°C.

3-(4, 5-dimethylthiazol-2-yl)-2, 5-diphenyltertrazolium bromide tetrazolium (MTT), Hoechst 33342, propidium iodide (PI), RNase A, N-acetylcysteine (NAC), and pifithrin-α were purchased from the Sigma-Aldrich. Guava Nexin Reagent was obtained from Millipore Corporation (Billerica, MA, USA). 2’, 7’-dichlorofluorescein-diacetate (DCFH-DA) was from Molecular Probes Inc. (Eugene, OR, USA). The aspartate aminotransferase (AST), alanine aminotransferase (ALT), blood urea nitrogen (BUN), and serum creatinine (Cr) assay kit were provided by Nanjing Jiancheng Bioengineering Institute (Nanjing, China).

### Cell lines

The human colorectal cancer SW-480, SW-620, Caco2 cells and human normal umbilical vein endothelial cell HUVEC were obtained from the Cell Bank of the Chinese Academy of Science (Shanghai, China). Cells were cultured in RPMI-1640, L-15 medium (Sigam-Aldrich) or Dulbecco’s modified Eagle’s medium (DMEM, Gibco, Life Technologies, USA) supplemented with 10% fetal bovine serum (FBS, Hyclone, USA), 100 U/ml penicillin, 100 μg/ml streptomycin, and 1 mM glutamine. Cultures were maintained at 37°C with humidity and 5% CO_2_.

### Cell viability assay

Cell viability was evaluated using MTT assay and colony formation test. For MTT assay, cells (1 × 10^5^ cells/ml) were seeded in 96-well plates (Corning Inc., NY, USA) overnight and exposure to 5-Fu (1, 5, 10, 50, 100, 300 μg/ml), Gyp (70, 85, 100 μg/ml) or 5-Fu + Gyp for 24 and 48 h (solvent control exposure to 80% ethanol and DMSO simultaneously). After treatment, the cell viability was determined by adding 10 μl MTT solution (5 mg/ml in PBS) to each well followed by incubation for 4 h at 37°C with 5% CO_2_. The MTT mixture was removed and 150 μl DMSO was added to each well. Samples were agitated on a shaker for 15 min, and the absorbance at 570 nm was recorded using a micro-plate reader (Bio-Tek, ELX800, USA). Cell viability was calculated as follows: (1—average absorbance of treated group/average absorbance of control group) × 100%.

Colony formation test was performed to evaluate the long-term proliferative potential of SW-480 or Caco2 cells following 5-Fu and / or Gyp treatment. Cells were seeded in 6-well plates at a density of 1000 cells/well and cultured for 7–10 days at 37°C with 5% CO_2_. The medium was changed every 3 days until visible colonies formed. Then the colonies were fixed with 4% paraformaldehyde at 4°C for 15 min and stained using Giemsa for 30 min. The samples were washed with PBS and dried out at room temperature. The number of stained colonies that contained 50 cells was manually counted. Proliferation potential was calculated as follows: relative colony formation rate (%) = number of colonies in the treatment group/number of colonies in the control group × 100%.

### Evaluation for combination index

After detection the single and combination inhibitory effects of 5-Fu and Gyp, the combination index (CI) was calculated according to the method of Chou and Talalay [21] using CalcuSyn software program (Biosoft, Cambridge, UK). The value of CI is a quantitative measure of the degree of interaction between drugs. CI < 1, indicates synergism;CI = 1, denotes additive effects; CI > 1, denotes antagonism.

### Determination of cell apoptosis

Cell apoptosis was detected using Guava Nexin assay, which utilizes Annexin V-PE to detect the phosphatidylserine on the external membrane of apoptotic cells [22]. SW-480 cells (2 × 10^5^ cells/ml) were seeded in 24-well plates and treated with 5-Fu and / or Gyp for 24 h, then cells were harvested and washed with PBS. After that 100 μl cells of each sample was suspended in a mixture of 100 μl Annexin V-PE and 7-AAD binding buffer, incubated at 37°C for 20 min in the dark and subjected to flow cytometry analysis (Millipore, USA) immediately. Histograms were analyzed using FCS Express V3 software.

### Hoechst 33342 staining

Hoechst 33342 staining was used to confirm the alterations of nuclei morphology of SW-480 cells after 5-Fu, Gyp or 5-Fu + Gyp treatment. Briefly, after incubation for 24 and 48 h, cells were stained with 10 mM Hoechst 33342 at 37°C in the dark for 15 min, then washed three times with PBS and observed using a fluorescence microscopy with standard excitation filters (Nikon, Japan).

### Cell cycle analysis

SW-480 cells were seeded in 24-well plates and exposure to 5-Fu, Gyp or 5-Fu + Gyp for 24 and 48 h. Then the treated cells were harvested and disposed as following steps: washed twice with cold PBS, fixed with 70% ice-cold ethanol at -20°C overnight, washed twice with cold PBS, incubated with 100 mg/ml RNase A for 30 min at 37°C, after that stained with 50 mg/ml PI in the dark for 30 min and subjected to flow cytometry analysis. For each experiment, 5,000 cells were recorded. The obtained results were analyzed by the Cell Quest software.

### Assessment of DNA damage

PI intercalates in DNA and the size of DNA fragments appears as a hypoploid DNA histogram [23]. Cells in 24-well plates were treated with 5-Fu, Gyp or 5-Fu + Gyp for 24 and 48 h, then stained with 5 μg/ml PI, permeabilized by freeze-throwing and subjected to flow cytometry analysis. Histograms were analyzed using FCS Express V3 software.

Comet assay, a sensitive and rapid technique for detection of DNA damage in individual cells, was performed as previously described [24].

### Measurement of intracellular ROS production

The change of intracellular ROS level was measured using the oxidative conversion of the sensitive fluorescent probe 2’, 7’-dichlorofluorescein-diacetate (DCFH-DA) to fluorescent 2’, 7’-dichlorofluorescein (DCF). DCFH-DA readily diffuses through the cell membrane and is hydrolyzed enzymatically by intracellular esterases to form non-fluorescent DCFH, which is then rapidly oxidized to form highly fluorescent DCF in the presence of ROS, and the fluorescence intensity is proportional to ROS production. Cells were incubated with 5-Fu, Gyp or 5-Fu + Gyp for 6 and 12 h, then stained by 10 μM DCFH-DA at 37°C in the dark for 30 min. After washing with PBS three times, cells were viewed under a fluorescence microscope, immediately (Nikon, Japan).

NAC (5 mM) was used as a ROS scavenger added to culture medium simultaneously with 5-Fu and / or Gyp adding. The ROS generation, DNA damage and cell apoptosis were analyzed as described above.

Pifithrin-α (10 μM) was used as an inhibitor of p53 to diminish the expression of p53 to culture medium when cells were incubated with 5-Fu and / or Gyp. The cell viability, ROS generation and DNA damage were analyzed as described above.

### Western blotting analysis

Western blotting was performed as previously described [25]. Proteins (60 μg) were electrophoresed on 5–13.5% polyacrylamide gels, and the gels were transferred onto nitrocellulose membranes (Millipore, MA, USA) that were incubated with relevant antibodies. The following antibodies were used: Phospho-p53 (Ser15), Phospho-cdk2 (Thr160), Caspase 3, Caspase 9, Bcl-2, Ser139-Histone H2A.X, Cleaved-PARP, β-actin (Cell Signaling Technology, Danvers, MA, USA) and Cyclin E (Santa Cruz Biotechnology, Santa Cruz, CA, USA). The β-actin expression was used as reference band. The protein expression rate was quantified by Quantity One software.

### Xenograft tumor model

The murine colorectal cancer CT-26 cells was obtained from the Cell Resource Center, Institute of Basic Medical Science (Beijing, China), and the condition of culture was same with SW-480 cells. The BALB/c mice (female, 18–20 g body weight) were supplied by the Experimental Animal Center of the Fourth Military Medical University (Xi’an, China). They were housed in an air-conditioned room at 23 ± 2°C, with free access to food and water and were maintained on a 12 h light-dark cycle. After being fed in our facility for 1 week, expect three mice were used as normal control, all others were inoculated with 0.1 ml of CT-26 cells (1 × 10^7^ cells/ml) into the left oxter region and randomly divided into 6 groups with 6–8 animals in each group. Treatment initiated on the day after CT-26 cells implantation (day 1). Group I was given reverse osmosis water via gastric lavage daily and regular saline via intraperitoneally injected every other day as the control group; Group II was injected with 5 mg/kg 5-Fu intraperitoneally every other day; Group III was given 25 mg/kg Gyp via gastric lavage every day; Group IV was given 50 mg/kg Gyp via gastric lavage every day; Group V was given 5 mg/ml 5-Fu + 25 mg/ml Gyp and Group VI was given 5 mg/kg 5-Fu + 50 mg/kg Gyp. The long (a) and short (b) diameters of the tumors were measured using slide calipers every day after Day 6 for calculated tumor volume as the formula: ab^2^/2. Body weight was also measured.

Mice were observed daily for clinical signs and sacrificed on the nineteenth day. Tumor specimens were weighed and fixed with 10% formalin for at least 24 h, embedded with paraffin, then stained by hematoxylin-eosin staining and immunohistochemistry. The inhibition rate was calculated as follows: (1- average of tumor weight of the treated group/average tumor weight of the control group) × 100%. Meanwhile, spleen were excised and weighed for calculated the spleen indexes as follows: spleen weight/body weight × 100%. The levels of AST, ALT, BUN and Cr in the serum were also detected.

Experiments using mice were performed in accordance with the National Institute of Health’s Guide for the Care and Use of Laboratory Animals and were approved by the university’s Institutional Animal Care and Use Committee of Shaanxi Normal University (Xi’an, China).

### Immunohistochemistry assay

Immunohistochemistry was performed on 7 μm sections of paraffin-embedded specimens using monoclonal anti-PCNA antibody (Abcam, Cambridge, UK). Briefly, after deparaffinization and hydration, the sections were treated with heat-mediated antigen retrieval using 10 mM citrate buffer (PH 6.0) for 15 min and permeabilized with 0.2% Triton X-100 for 15 min. Then the endogenous peroxidase activity was quenched by 3% hydrogen peroxide solution for 10 min. Non-specific binding was prevented by incubation with 5% normal goat serum for 15 min. After that, the sections were incubated with anti-PCNA antibody (1:8000 dilutions) overnight at 4°C in a moist chamber. Antibody binding was detected using horseradish peroxidase-conjugated secondary antibody at 37°C for 30 min. Then sections were visualized by diaminobenzidine solution, counterstained lightly with hematoxylin, dehydrated with ethanol and observed using light microscopy (Nikon, Japan).

### Statistical analysis

SPSS 16.0 software (SPSS Inc., Chicago) was used for statistical analysis. Values are expressed as mean ± standard deviation (SD) of three samples obtained from three independent experiments. Statistical comparisons were made using one-way analysis of variance (ANOVA), p* < 0.05 was considered to be significant difference, p** < 0.01 and *p* ^*##*^ < 0.01 were considered to be extremely significant difference.

## Results

### Gyp synergistically potentiates 5-Fu-induced cell viability inhibition

Fig 1B shows the cytotoxicity of 5-Fu on SW-480, SW-620 and Caco2 cells for treatment 48 h respectively. Caco2 cells performed more sensitive than SW-480 and SW-620 cells to 5-Fu treatment (the IC50 value was calculated as 31.75 μg/ml for Caco2 and 257.23, 366.40 μg/ml for SW-480, SW-620 cells). When the concentration exceed 50 μg/ml, SW-480 and SW-620 cell show somewhat resistance to 5-Fu treatment. However, Gyp inhibited SW-480,SW-620 and Caco2 cell proliferation in a dose- and time-dependent manner (the IC50 value was calculated as 99.59, 107.53 and 112.22 μg/ml for SW-480, SW-620 and Caco2 cells after incubation for 24 h, Fig 1C–1E). When co-treated cells with Gyp (70, 85, 100 μg/ml) and 5-Fu (5, 10 μg/ml) simultaneously, the combined treatment even in the low doses displayed much higher anti-proliferative activities on SW-480, SW-620 and Caco2 cells than that of single treatment. For instance, treatment of SW-480 cells with 5 μg/ml 5-Fu or 70 μg/ml Gyp alone for 24 h inhibited cell viability by 14.6 ± 2.67% (*p* ˂ 0.05 *vs* control) and 13.8 ± 2.80% (*p* ˂ 0.05 *vs* control), respectively. Nevertheless, treatment of SW-480 cells with 5 μg/ml 5-Fu + 70 μg/ml Gyp for 24 h significant inhibited cell viability by 53.29 ± 1.02% (*p* ˂ 0.01 *vs* control and 5-Fu alone). When the incubation time was increased to 48 h, the cell viability inhibition of 5 μg/ml 5-Fu was 32.37 ± 3.22% (*p* ˂ 0.01 *vs* control), 70 μg/ml Gyp was 24.87 ± 1.83% (*p* ˂ 0.01 *vs* control), 5 μg/ml 5-Fu + 70 μg/ml Gyp was 72.11 ± 1.53% (*p* ˂ 0.01 *vs* control and 5-Fu alone).

To further validate the synergistic feature of 5-Fu and Gyp, the cell viability inhibition effects of the single and combined treatment were analyzed using CalcuSyn software. The CI value for 5-Fu + Gyp treatment SW-480 cells was 0.68 ± 0.05, SW-620 cells was 0.65 ± 0.10 and Caco2 cells was 0.72 ± 0.07 under the applied dosages (Fig 1C–1E). For SW-480 cells, the combination of 5-Fu (5 μg/ml) and Gyp (70 μg/ml) displayed the best synergistic inhibition capacity (the CI values were 0.63 and 0.68 for 24 and 48 h), which was selected for further mechanistic studies. Moreover, 5-Fu and Gyp in combination exhibited much weaker toxicity toward human normal umbilical vein endothelial cell HUVEC (Fig 1F).

Colony formation test was performed to further verify the proliferative potential of SW-480 and Caco2 cells after 5-Fu and / or Gyp treatment. Fig 1G showed that 5-Fu, Gyp, 5-Fu + Gyp all inhibit long-term proliferative potential of SW-480 or Caco2 cells, in comparison to untreated group (*p* < 0.01 vs control), and the most significant one with fewer colons formation were observed in 5-Fu + Gyp group (*p* < 0.01 vs 5-Fu or Gyp alone).

### Apoptotic response triggered by 5-Fu and / or Gyp

**Fig 2:**
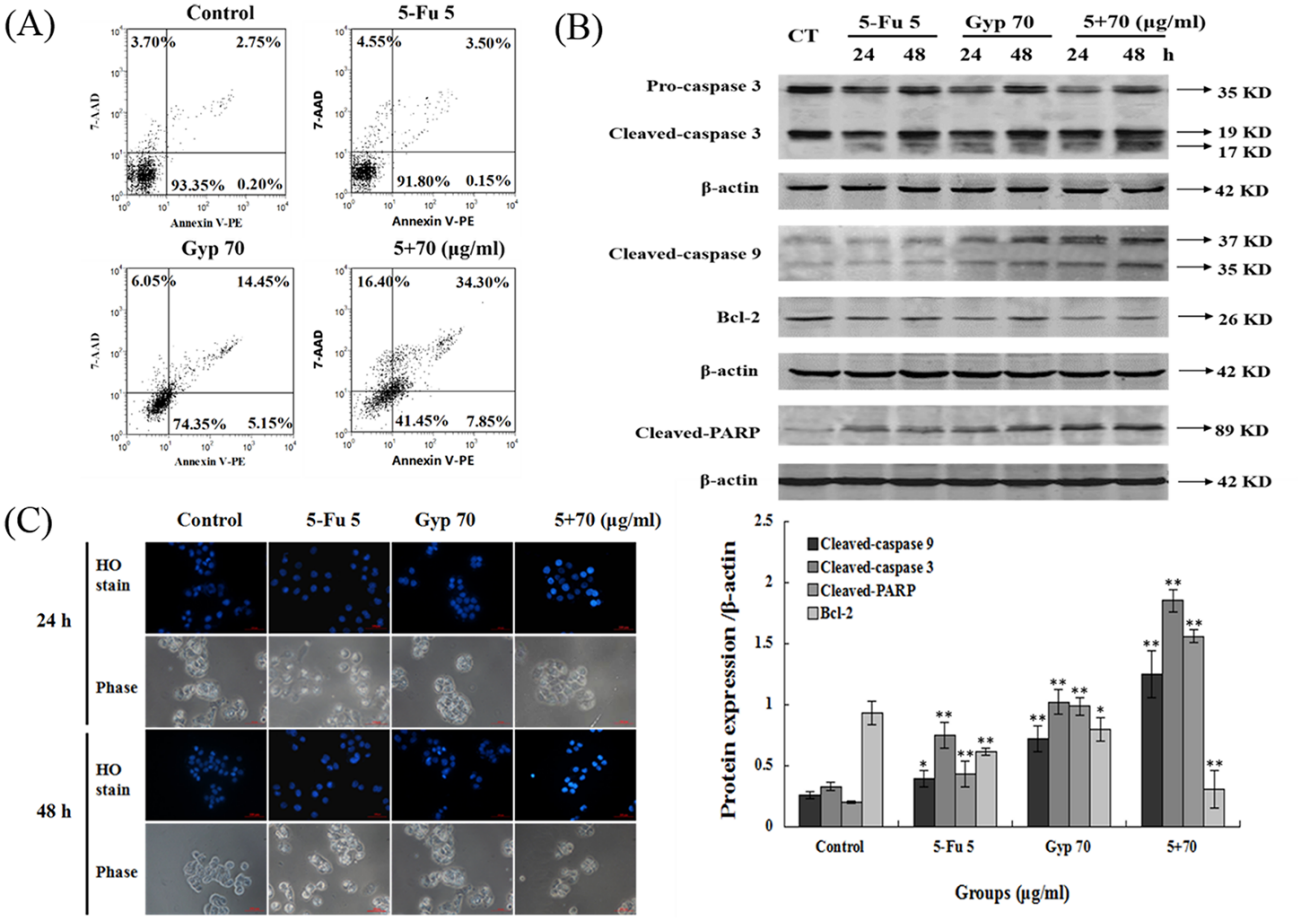
Apoptotic response induced by 5-Fu and Gyp. (A) Cell apoptosis was detected by Annexin V-PE/7-AAD assay after 5-Fu and / or Gyp treatment for 24 h in SW-480 cells. (B) The expression level of caspase 3, caspase 9, Bcl-2 and cleaved-PARP in SW-480 cells was detected by western blotting. (C) Nuclear condensation and cell morphology change in SW-480 cells after 5-Fu co-treated with Gyp was observed using Hoechst 33342 staining. Experiments were done independently in triplicate per experimental point, and representative results were shown. All data are expressed as means ± SD of triplicates and p* < 0.05, p** < 0.01 versus control.

As shown in Fig 2A, in untreated control, 93.35 ± 0.84% were viable, 0.2 ± 0.06% cell population were in the early stage of apoptosis (lower right) and 2.75 ± 1.32% cell population were in the late stage of apoptosis (upper right). In 5-Fu treatment group, the apoptotic cell population (lower right and upper right) were 3.65 ± 0.81%. In Gyp treatment group, 20.6 ± 1.63% cells were in the stage of apoptosis (*p* < 0.01 *vs* control). While in the 5-Fu + Gyp group, 42.15 ± 0.67% cells were in the apoptotic stage (*p* < 0.01 *vs* control and 5-Fu alone).

Western blotting was performed to analysis the potential mechanism worked in 5-Fu and Gyp triggered apoptosis. As shown in Fig 2B, 5-Fu + Gyp greatly enhanced caspase 3, caspase 9 and PARP cleavage and down-regulated the expression of anti-apoptotic protein Bcl-2 in SW-480 cells (*p* < 0.01 or *p* < 0.05 *vs* control).

### Morphological changes induced by 5-Fu and Gyp on SW-480 cells

Fig 2C shows the morphological changes after 5-Fu and / or Gyp treatment using Hoechst 33342 staining. Slightly blue and homogeneous cells were observed in control group; 5-Fu or Gyp treated cells showed slight enhancement of Hoechst 33342 staining; while in 5-Fu + Gyp group, cells exerted remarkably changes: condensed chromatin, fragmented punctuate blue nuclear fluorescence. In addition, the phase images revealed that cells in 5-Fu + Gyp treatment group were shrunken to abnormal round type, and the cell numbers were also reduced distinctly.

### Effects of 5-Fu and / or Gyp on cell cycle distribution

**Fig 3:**
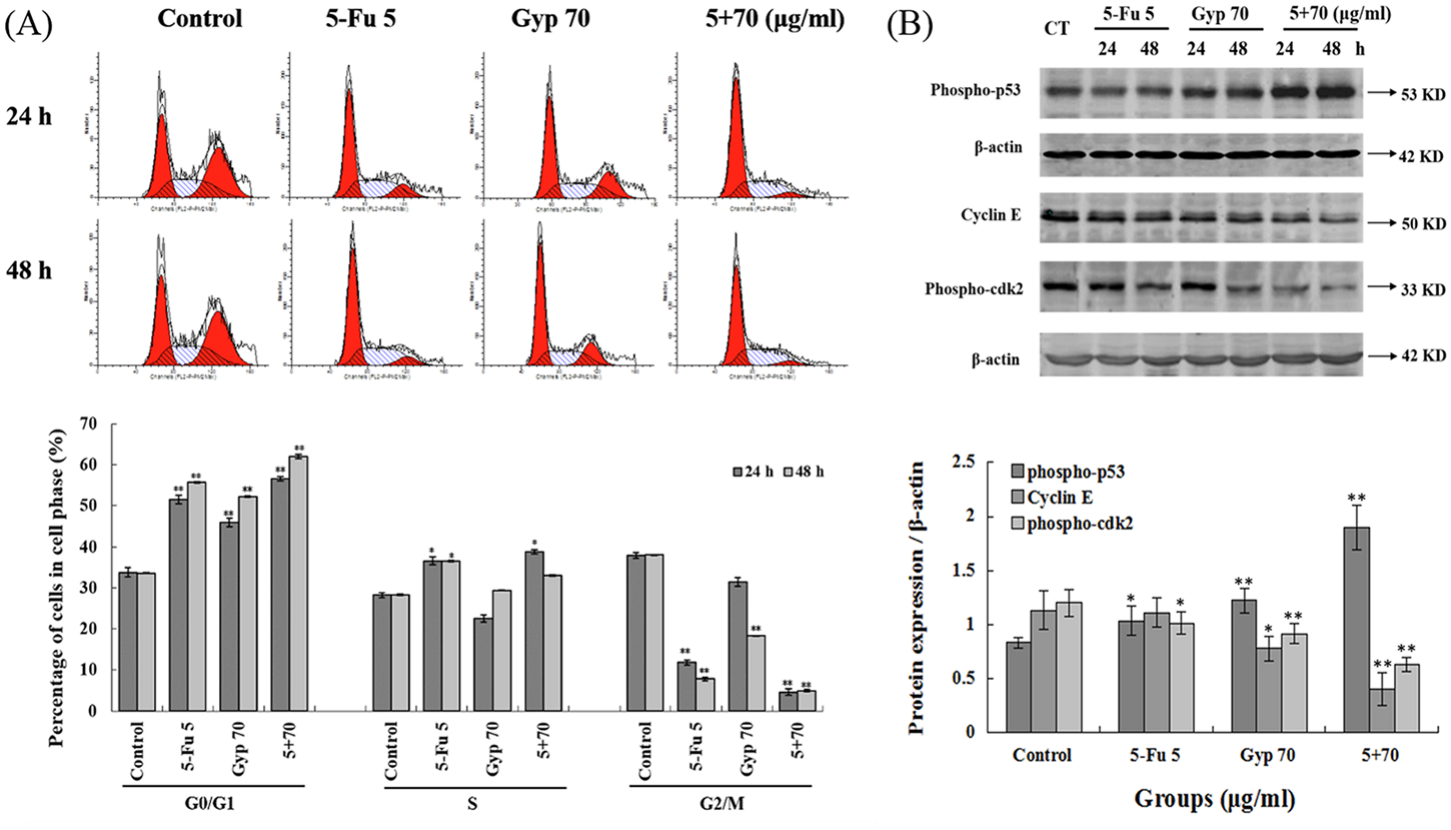
Effects of 5-Fu and / or Gyp on cell cycle distribution. (A) Cell cycle distribution after 5-Fu and Gyp treatment. (B) The expression of phospho-p53, phospho-cdk2 and cyclin E after 5-Fu and / or Gyp treatment in SW-480 cells were detected by western blotting. Experiments were done independently in triplicate per experimental point, and representative results were shown. All data are expressed as means ± SD of triplicates and p* < 0.05, p** < 0.01 versus control.

Flow cytometry analyse in Fig 3A indicate that compared with control (G0/G1-phase, 33.8 ± 1.06%; S-phase, 28.25 ± 0.59%, and G2/M-phase, 37.94 ± 0.65%), there was an accumulation of cell population in G0/G1-phase (51.56 ± 1.08%, *p* < 0.01 *vs* control) and S-phase (36.6 ± 0.91%, *p* ˂ 0.05 *vs* control) after 5-Fu treatment. Gyp induced an accumulation of cell population in G0/G1-phase (45.97 ± 1.06%, *p* < 0.01 *vs* control). In the 5-Fu + Gyp treatment group, there were more cells in the G0/G1-phase (56.58 ± 0.57%, *p* < 0.01 *vs* control) and S-phase (38.78 ± 0.46%, *p* < 0.05 *vs* control). A similar trend was also found after 5-Fu, Gyp or 5-Fu + Gyp treatment for 48 h.

The expression of phospho-p53, phospho-cdk2 and cyclin E in SW-480 cells after 5-Fu and / or Gyp treatment were detected by western blotting. As shown in Fig 3B, 5-Fu + Gyp remarkably increased the expression of phospho-p53, but decreased the expression of cyclin E and phospho-cdk2 (*p* < 0.01 or *p* < 0.05 *vs* control) after 24 and 48 h incubation.

### DNA damage response induced by 5-Fu, Gyp and 5-Fu + Gyp

**Fig 4:**
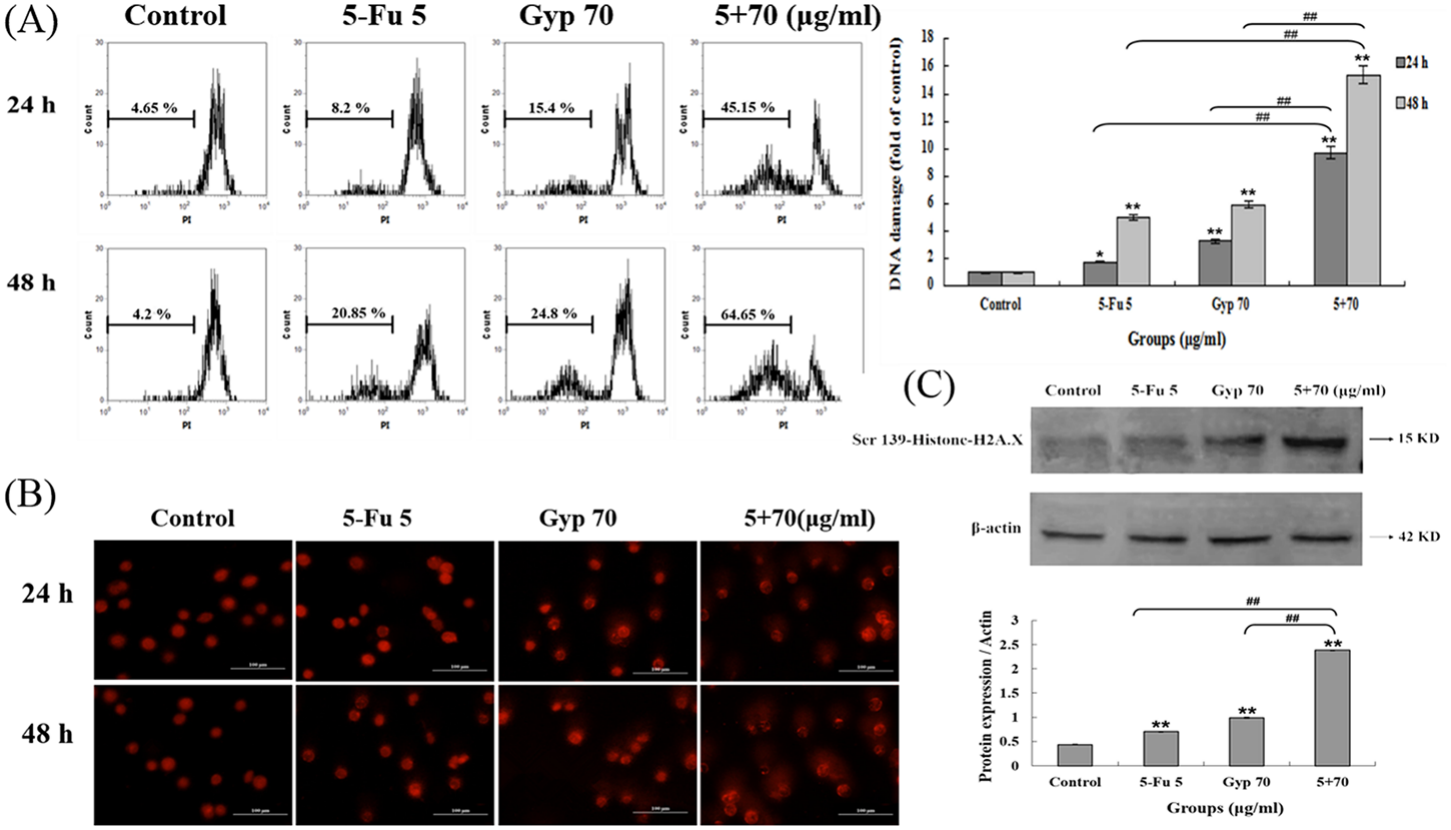
DNA response induced by 5-Fu, Gyp and 5-Fu + Gyp. (A) DNA fragmentation was detected using flow cytometry after 5-Fu, Gyp and 5-Fu + Gyp treatment in SW-480 cells after 24 and 48 h. Histograms showed number of cell channels (vertical axis) vs. PI fluorescence (horizontal axis). (B) Comet assay was performed for detection of DNA damage after 5-Fu co-treated with Gyp. (C) The expression level of Ser139-Histone H2A.X was determined using western blotting. Experiments were done independently in triplicate per experimental point, and representative results were shown. All data are expressed as means ± SD of triplicates and p* < 0.05, p** < 0.01 versus control; *p* ^*##*^ < 0.01 versus 5-Fu or Gyp alone group.

As described in the methods, flow cytometry analysis was performed to investigate the efficacy of 5-Fu + Gyp treatment on DNA fragmentation in SW-480 cells. Fig 4A shows that the level of DNA fragmentation in control group was 4.66 ± 1.55%. After being treated by 5-Fu, Gyp or 5-Fu + Gyp for 24 h, there was an increasing trend (8.2 ± 0.48%, 15.4 ± 2.74%, and 45.15 ± 1.63%, respectively) on DNA fragmentation levels. The degree of DNA fragmentation in 5-Fu + Gyp treated group was significant increased (*p* < 0.01 *vs* control and 5-Fu alone). A similar phenomenon was found after 48 h incubation and the DNA fragmentation was increased to 64.65 ± 3.62% (*p* < 0.01 *vs* control and 5-Fu alone) after 5-Fu + Gyp treatment when the DNA fragmentation in control, 5-Fu, and Gyp alone group were 4.2 ± 0.81%, 20.85 ± 2.70% and 24.80 ± 1.96%, respectively.

Comet assay and the expression of γH2A.X were performed to further verify that DNA damage was aggravated by 5-Fu combined with Gyp. Fig 4B shows that 5-Fu, Gyp and 5-Fu + Gyp all induced varying degrees of DNA damage in comparison with untreated control group, and the most significant one with more cells produced a large tail of comet was observed in 5-Fu + Gyp group. Fig 4C shows that 5-Fu combined with Gyp aggravated the phosphorylation of γH2A.X.

### ROS accumulation after 5-Fu + Gyp treatment

**Fig 5:**
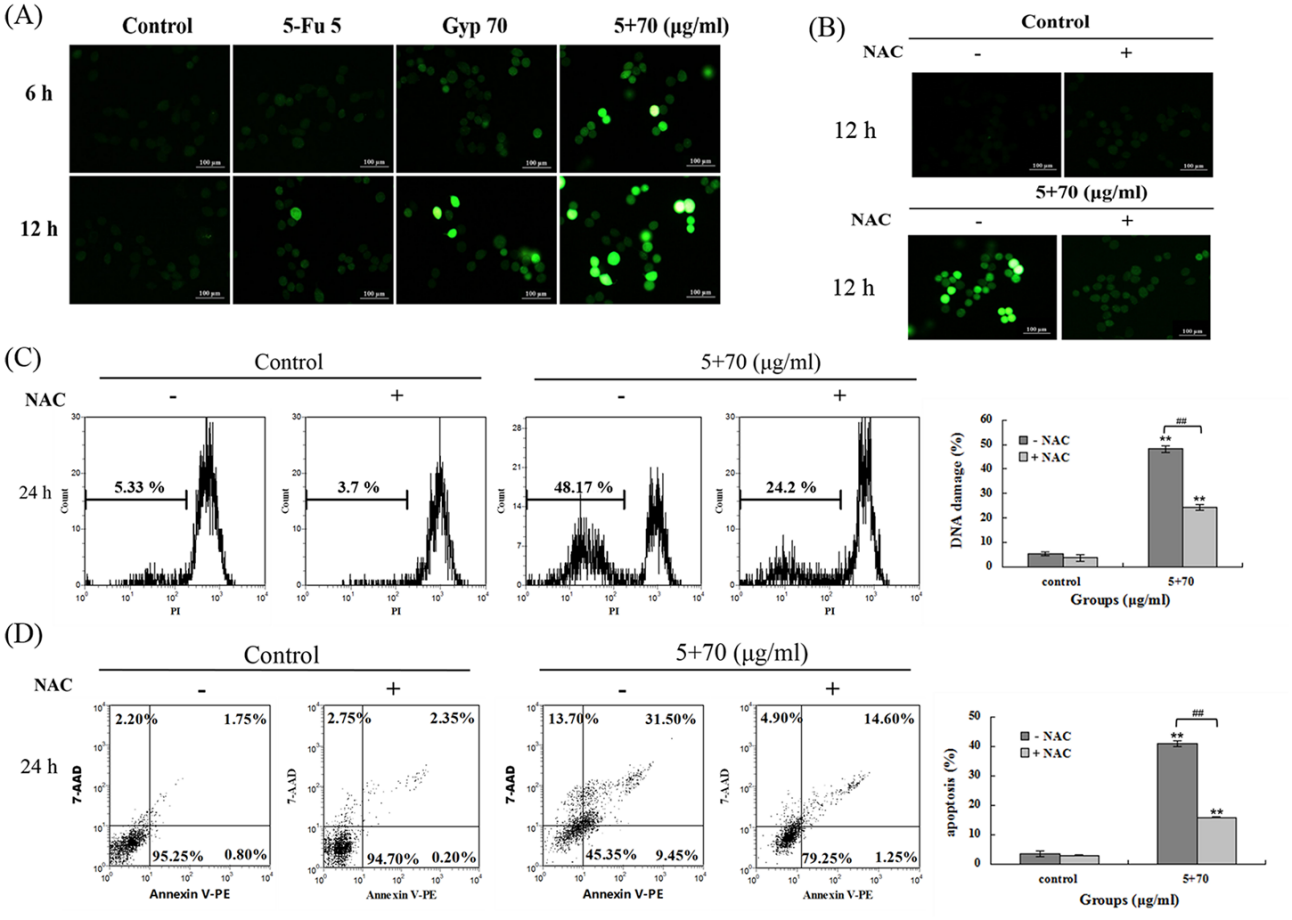
ROS accumulation after 5-Fu + Gyp treatment. (A) Intracellular ROS production after 5-Fu and / or Gyp treatment was detected using DCFH-DA staining. (B-D) Intracellular ROS production, DNA fragmentation and cell apoptosis in SW-480 cells were measured after added NAC to culture medium simultaneously with 5-Fu and Gyp. Experiments were done independently in triplicate per experimental point, and representative results were shown. All data are expressed as means ± SD of triplicates and p** < 0.01 versus control; *p* ^*##*^ < 0.01 versus 5-Fu + Gyp group.

Intracellular ROS was detected by DCFH-DA staining at 6 and 12 h after different treatments. As Fig 5A illustrated, compared to control cells in 5-Fu + Gyp group showed strong DCF fluorescence at 6 and 12 h post treatment, cell in Gyp group showed some visible DCF fluorescence at 6 h and slightly enhanced at 12 h, while no obvious fluorescence was observed in 5-Fu alone group. To further validate the role of ROS in the anti-tumor effect of 5-Fu + Gyp, NAC was added to diminish the ROS level, then DNA damage and cell apoptosis were analyzed. Results showed that NAC co-treatment effective suppressed the intracellular ROS production with the DCF fluorescence vanished (Fig 5B). The DNA damage caused by 5-Fu + Gyp treatment was partially rescued by NAC (*p* < 0.01 *vs* control, Fig 5C). 5-Fu + Gyp caused cell apoptosis was also significantly prevented by NAC (*p* < 0.01 *vs* control, Fig 5D).

### Role of p53 in 5-Fu + Gyp treatment

**Fig 6:**
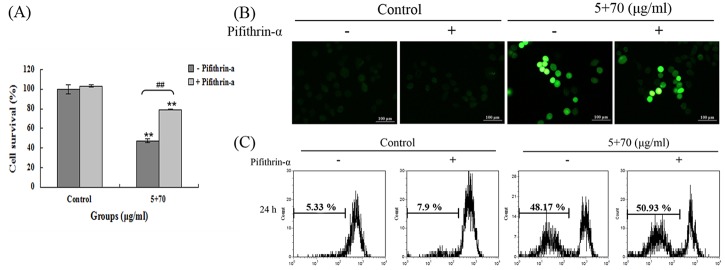
Role of p53 in 5-Fu + Gyp treatment. (A) Cell viability, (B) Intracellular ROS production and (C) DNA damage were detected after added pifithrin-α to culture medium simultaneously with 5-Fu plus Gyp. Experiments were done independently in triplicate per experimental point, and representative results were shown. All data are expressed as means ± SD of triplicates and p** < 0.01 versus control; *p* ^*##*^ < 0.01 versus 5-Fu + Gyp group.

The excessive production of ROS can damage DNA and provoke p53 activity to induce cell cycle arrest and apoptosis [26,27]. Our results found p53 phosphorylation was significantly increased after 5-Fu + Gyp treatment (Fig 3B). To further investigate the role of p53 in 5-Fu co-treated with Gyp-induced apoptosis, pifithrin-α, an inhibitor of p53, was added to diminish the expression of p53, and then intracellular ROS level, DNA damage and cell viability were detected. Fig 6A suggests that pifithrin-α remarkably reversed the 5-Fu + Gyp-induced cell death in SW-480 cells with the cell viability was increased from 47.49 ± 2.1% to 79.22 ± 0.63% (*p* < 0.01 *vs* control). However, co-treatment with pifithrin-α do not influence the intracellular ROS accumulation and severe DNA damage caused by 5-Fu + Gyp treatment (Fig 6B and 6C).

### Anti-tumor effect in vivo

**Fig 7:**
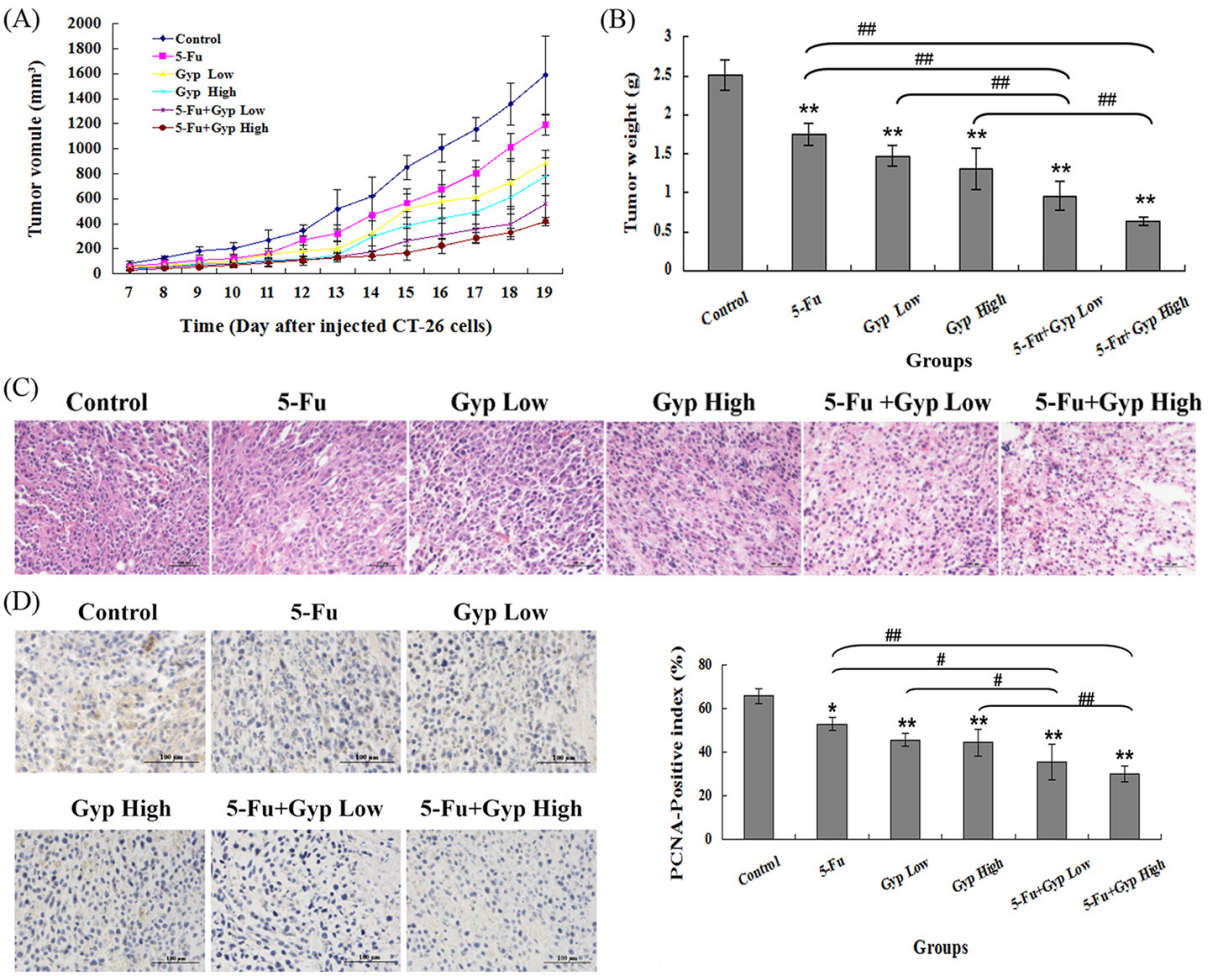
Anti-tumor effect of 5-Fu and / or Gyp on CT-26-colon-carcinoma-bearing mice. (A) The growth of tumor volume in different groups were monitored every day after inoculated with CT-26 cells for seven days. (B) The final tumor weight in each group after treatment. (C) The microstructure of tumor tissue in different groups was observed by Hematoxylin-eosin staining. (D) The expression level of PCNA after different treatment was performed using immunohistochemistry detection and quantified values shown are the average percentage of positive nuclei. All data are expressed as means ± SD of triplicates and p* < 0.05, p** < 0.01 versus control; *p* ^*##*^ < 0.01 versus 5-Fu or Gyp alone group.

CT-26 xenograft tumor mouse model was employed to investigate the *in vivo* anti-tumor efficacy of 5-Fu and Gyp. None of the mice died over the course of treatment and all mice developed tumor xenograft, successfully. On the 19^th^ day post treatment, the mean tumor volume in (1) control, (2) 5 mg/kg 5-Fu, (3) 25 mg/kg Gyp, (4) 50 mg/kg Gyp, (5) 5mg/kg 5-Fu + 25 mg/kg Gyp and (6) 5mg/kg 5-Fu + 50mg/kg Gyp treatment group were 1,589.49 ± 312.192 mm^3^, 1192.95 ± 80.55 mm^3^, 866.79 ± 103.11 mm^3^, 777.182 ± 153.89 mm^3^, 560.77 ± 158.325 mm^3^ and 420.351 ± 32.593 mm^3^, respectively (Fig 7A). The mean tumor weights in different groups were also calculated. And results showed that the tumor weight inhibition rates were 29.97%, 41.45%, 48.07%, 61.65% and 74.43% for 5 mg/kg 5-Fu, 25 mg/kg Gyp, 50 mg/kg Gyp, 5mg/kg 5-Fu + 25 mg/kg Gyp and 5mg/kg 5-Fu + 50mg/kg Gyp treatment group, respectively (Fig 7B). The trend of tumor weight inhibition was consistent with tumor volume inhibition.

### Histological and immunohistochemical analysis of tumor sections

Hematoxylin-eosin staining was used to observe the microstructure changes after different treatment. The control group showed compact tumor cells with blue-purple nuclei and pink cytoplasm. In the 5-Fu or Gyp treatment groups, the tumor cells were sparse and separated from each other. In 5-Fu + Gyp treatment groups, the structure of tumor tissue was more serious damaged than that in 5-Fu or Gyp alone groups accompanied by obvious vacant sections and lots of nuclear fragments (Fig 7C).

The expression level of PCNA was performed to evaluate the anti-proliferation effect of 5-Fu and Gyp *in vivo*. In comparison to control group, the percentage of PCNA-positive cells were reduced in 5-Fu or Gyp treatment group (p < 0.01 *vs* control) and have a further decrease in the combined 5-Fu + Gyp treatment groups (*p* < 0.01 *vs* control, 5-Fu alone and Gyp alone, Fig 7D).

### Toxicity evaluation of the combination 5-Fu + Gyp

**Fig 8:**
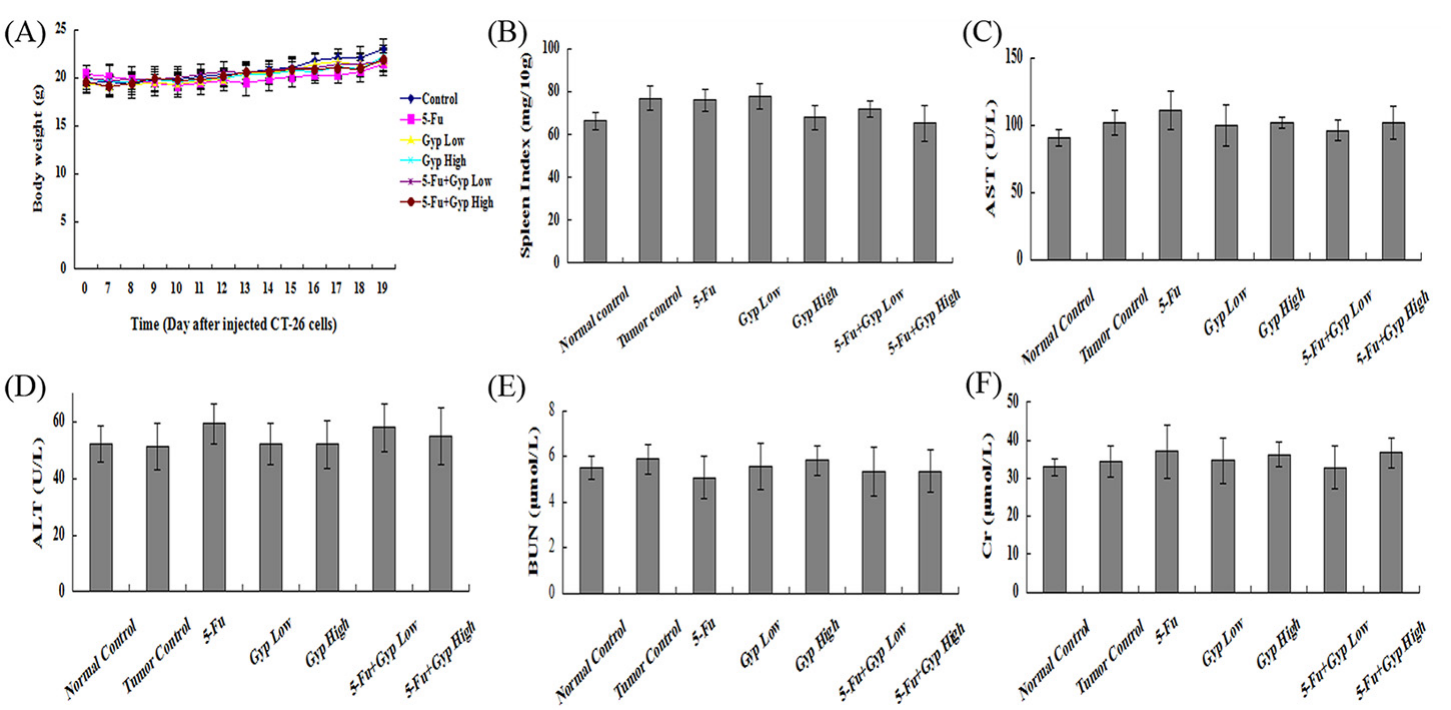
Toxicity evaluation of combination treatment. (A) Body weights were recorded every day in different groups every day after inoculated with CT-26 cells for seven days. (B) Spleen indexes were calculated after various treatment. (C-F) The level of AST, ALT, BUN and Cr in the serum were detected. All data are expressed as means ± SD of triplicates.

One of the main concerns in developing new cancer therapy is the unknown toxicity. At the end of experiment, the mice were necropsied. Body weights in each group showed slight increase (Fig 8A). There was no significant difference of the spleen index among the control group and other treated groups (Fig 8B). Moreover, no clear metastasis and hemorrhages, or injury to the liver and kidney was visible to the naked eye. The levels of AST (Fig 8C), ALT (Fig 8D), BUN (Fig 8E) and Cr (Fig 8F) in the serum also did not show differences among distinct groups.

## Discussion

Because of the limited efficiency of single-agent chemotherapy in the treatment of colorectal cancer, many combination therapies, by using valid chemo-sensitizers to augment the response rate of existing anti-cancer drug and simultaneously overcome their resistance and undesired reaction have been applied in the clinic recently [5,9,28]. Numerous studies have reported that extracts of traditional Chinese medicine, such as resveratrol, curcumin, ginsenoside could act as chemo-sensitizer to enhance the efficacy of 5-Fu in CRC cells [9,29,30]. In this study, we show that Gyp displays a splendid chemo-sensitization effect to potentiate the 5-Fu-induced cell growth inhibition *in vitro* and *in vivo*, suggesting Gyp can reduce the dose of 5-Fu needed to achieve desired effects and then avoid the high dosage-induced drug resistance and undesired reaction.

5-Fu can exert its cytotoxic effect by inducing DNA damage, inhibiting cell cycle arresting and leading to cell apoptosis [4,31]. Gyp, the main components isolated from *Gynostemma pentaphyllum*, has reported to trigger apoptosis in colorectal cancer cells through the mitochondria-dependent pathway [17–19]. The MTT assay and colony formation test demonstrated that the cell viability of SW-480,SW-620 and Caco2 were significantly decreased after co-treated with a low doses of 5-Fu and Gyp, in comparison to either single treatment (Fig 1C–1G). Moreover, 5-Fu and Gyp in combination exhibited superior anti-tumor effect on CT-26 xenograft mouse model, displaying more efficient inhibition of tumor volume and tumor weight (Fig 7A and 7B). The expression level of PCNA in tumor tissue were also decreased. (Fig 7D). These results suggest that 5-Fu + Gyp could synergistically suppress cancer cell proliferation and tumor growth.

Inducing tumor cell apoptosis is considered the most effective method for cancer therapy [32–34]. In this study, Gyp and 5-Fu in combination effectively induced SW-480 cell apoptosis (Fig 2A). Caspase 3, caspase 9, which are key components of apoptotic response [35], were also be activated after 5-Fu + Gyp treatment (Fig 2B), implies the occurrence of intrinsic mitochondria-mediated apoptotic pathway. These results were quite consistent with previous studies, which reveals that the activation of caspase cascade and mitochondrial-dependent pathways were involved in Gyp induced apoptosis in cancer cells [11, 36]. In addition, SW-480 cells after 5-Fu + Gyp treatment show specific morphological changes, such as membrane blebbing, cell shrinkage and chromatin condensation (Fig 2C), which further confirms that 5-Fu + Gyp had a synergistic role in induction of apoptosis in SW-480 cells.

5-Fu is an anti-cancer drug mainly targeting S-phase cells, and the metabolites of 5-Fu can only be incorporated into the DNA and RNA of cell cycling cells [3,4]. Prolonged G1 and S phase may provide 5-Fu with more time to induce DNA damage in cancer cells [37]. Studies reported that Gyp could inhibit cell cycle progression of tumor cells via blocking the transition from G1- to S-phase [15,17]. In our study, there was an accumulation of cell population in G0/G1-phase after Gyp treatment. And Gyp co-treatment significantly enhanced the 5-Fu-caused cell cycle arrest in G0/G1 and S-phase (Fig 3A). Western blotting indicates that 5-Fu + Gyp treatment notably increased the p53 phosphorylation, accompanied by cyclin E and cdk2 phosphorylation decreased in SW-480 cells (Fig 3B), reveals that Gyp could augment cell cycle arrest induced by 5-Fu and then enhance its anti-tumor activity.

**Fig 9:**
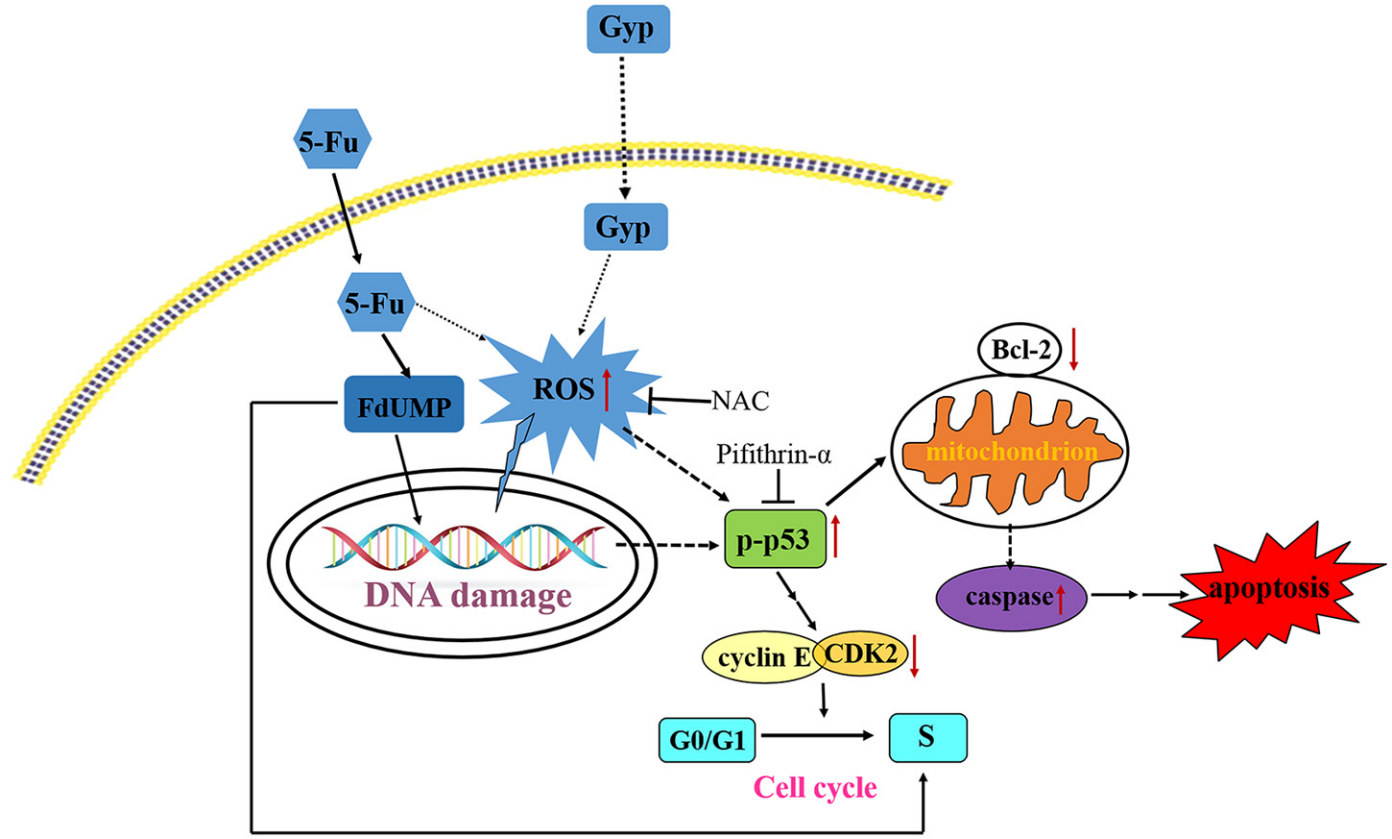
Proposed pathways of Gyp potentiates 5-Fu induced cell apoptosis and cell cycle arrest.

Current studies have supported the importance of ROS in therapeutic approaches to effectively kill cancer cells [26,38]. Excessive amounts of ROS can cause oxidative damage to lipids, proteins, and DNA, then leads to cell death [39,40]. Gyp could disturb the balance between ROS production and the cellular defense system, resulting in overproduction of ROS [41,42]. In this study, the intracellular ROS level and DNA damage response were significantly increased after Gyp or 5-Fu + Gyp treatment (Figs 4 and 5A). Co-treatment with NAC, a scavenger of ROS, the 5-Fu + Gyp-induced DNA damage and apoptosis were distinctly alleviated (Fig 5C and 5D), implying the important role of ROS in severe DNA damage and cell apoptosis after 5-Fu + Gyp in SW-480 cells. Moreover, we also used pifithrin-α, a specific of p53 inhibitor [43], to analysis the role of p53 in the anti-tumor effect of 5-Fu + Gyp. Results showed that pifithrin-α co-treatment did not affect ROS accumulation and DNA damage (Fig 6B and 6C), but rever cell growth inhibition in SW-480 cells (Fig 6A) after 5-Fu + Gyp treatment, illustrating that although p53 was not required for early oxidative DNA damage, p53 was critical for the occurrence of cell death in 5-Fu + Gyp treatment. Taken together, as a potential chemo-sensitizer, Gyp could dramatically enhance the 5-Fu-induced apoptosis and cell cycle arrest in SW-480 cells by triggering ROS-mediated DNA damage and activation of p53. The possible pathway of Gyp potentiates 5-Fu-induced proliferation inhibition of SW-480 cells may be proposed as in Fig 9. More in-depth studies are needed to support this proposal.

Finally, there were no detectable side effects after 5-Fu co-treated with Gyp treatment at the therapeutic dose in vivo according to the preliminary safety analysis (Fig 8), however, the present safety evaluation is somewhat limited and should be expanded by a series of rigorous assessments before clinically used.

## Conclusion

In summary, we performed both *in vitro* and *in vivo* experiments to assess the chemo-sensitization effect of Gyp to potentiate the anti-tumor effect of 5-Fu in colorectal cancer. Results suggested that Gyp displayed a splendid synergistic effect with 5-Fu to inhibit cancer cell proliferation and tumor growth. We propose that using 5-Fu and Gyp in combination could be as a highly efficient way to achieve anti-tumor synergism in the clinical treatment of colorectal cancer.
